# Draft Genome Sequence of Clostridium cadaveris Strain AGRFS2.2, Isolated from a Bovine Dairy Farm in New Zealand

**DOI:** 10.1128/MRA.00787-20

**Published:** 2020-08-27

**Authors:** Tanushree B. Gupta, Ruy Jauregui, Paul Maclean, Amila Srilal Nawarathana, Gale Brightwell

**Affiliations:** a Food Assurance Team, Hopkirk Research Institute, AgResearch, Palmerston North, New Zealand; b Knowledge and Analytics, Grasslands Research Centre, AgResearch, Palmerston North, New Zealand; University of Arizona

## Abstract

We report the draft genome sequence of a new Clostridium cadaveris strain, AGRFS2.2, isolated from soil in a bovine dairy farm environment in New Zealand. The genome is 3.6 Mbp long with a GC content of 31.3%. The genome sequence was found to be closely related to that of Clostridium cadaveris JCM 1392^T^.

## ANNOUNCEMENT

Clostridium cadaveris is a strictly anaerobic, motile, Gram-positive, rod-shaped, spore-forming bacterium that can be found in soil but mostly colonizes the gastrointestinal tract of animals and humans (1, 2). The bacterium was first isolated from putrefying bodies and was the most prominent species during the decay of cadavers—hence the name cadaveris (3). Mostly nontoxic and nonpathogenic in animals and humans, this bacterium has been associated with infections in humans (4–6). Here, we report the whole-genome sequence of a recently isolated Clostridium cadaveris strain, AGRFS2.2, isolated from a pasture soil sample from a bovine dairy farm in New Zealand’s Manawatu region.

Bacteria were isolated using a previously described methodology with slight modifications (7). Briefly, 10 g of soil was suspended in 50 ml of phosphate buffer (PB) and centrifuged at 3,466 × *g* for 1 h. The pellet was then suspended in 5 ml of PB and heated at 80°C for 10 min, and 1 ml of this was added to cooked meat glucose starch medium (8) and incubated anaerobically at 35°C for 48 h. The growth suspension was serially diluted, plated on Shahidi-Ferguson agar, and incubated anaerobically for 24 h (9). Each colony was further grown on sheep blood agar (SBA) to obtain isolated and pure colonies. Genomic DNA was extracted from these pure cultures grown in tryptic soy broth (Fort Richard Laboratories, New Zealand) using the phenol-chloroform extraction method (10). The quality and concentration of DNA were determined using a Qubit 2.0 fluorometer (Thermo Fisher Scientific, USA). Initial identification was conducted using 16S rRNA amplicon sequencing with forward primer pA 5′-AGAGTTTGATCCTGGCTCAG-3′ and reverse primer pH* 5′-AAGGAGGTGATCCAGCCGCA-3′ (11). The amplification method consisted of 93°C for 3 min, 92°C for 1 min, 55°C for 1 min, and 72°C for 2 min for 30 cycles followed by a final extension at 72°C for 3 min.

Genomic library preparation of *Clostridium* strain AGRFS2.2 was done with the NuGEN Celero DNA enzymatic library kit according to the manufacturer’s instructions. The library was sequenced using the Illumina MiSeq sequencing platform version 3 (Massey Genome Services, Palmerston North, New Zealand), producing 497,239 read pairs of 300 nucleotides (nt) and a total of 299,337,878 nucleotides, giving a coverage of 82.68×. The reads were quality trimmed, filtered, and assembled with the A5-miseq pipeline version 20160825 with default settings (12). The assembly produced 87 contigs with a total genome size of 3.6 Mb, an *N*_50_ value of 106 kb, and a GC content of 31.3%. A BUSCO version 3.0.2 (13) test using the bacterial reference produced a completeness score of 97.3% (144 complete sets, 0 duplicated, 0 fragmented, and 4 missing).

Analysis of 16S rRNA gene sequencing showed 100% sequence similarity between the new *Clostridium* strain AGRFS2.2 and *C. cadaveris* JCM 1392^T^. A two-way average nucleotide identity (ANI) test (http://enve-omics.ce.gatech.edu/ani/) was carried out (14), and due to the unavailability of the draft genome sequence of the *C. cadaveris* type strain, ANI scores were compared with those of other *C. cadaveris* isolates present in the NCBI database. ANI testing produced ∼99% value matching of the *Clostridium* strain AGRFS2.2 with other *C. cadaveris* isolates, indicating that it is possibly the same species; however, differences may lie in the strain level. *C. cadaveris* strains used for comparison were reported to be isolated from rumen fluid and human and pig gut, whereas *Clostridium* strain AGRFS2.2 was isolated from soil. Studies are required to sequence the whole genome of the type strain to cluster the available *C. cadaveris* strains appropriately. Further studies will be carried out to investigate the similarity or differences of the strain AGRFS2.2 with the type strain and the presence of any pathogenic traits.

As part of the submission process, NCBI annotated the genomic scaffolds with PGAP version 4.11 (15), resulting in 3,614 genes being annotated in total.

### Data availability.

The raw reads have been deposited in the NCBI SRA under the accession number PRJNA642910. This whole-genome shotgun project has been deposited at DDBJ/ENA/GenBank under the accession number JACATM000000000. The version described in this paper is version JACATM010000000.
